# Genome Sequence of *Lactobacillus curieae* CCTCC M 2011381^T^, a Novel Producer of Gamma-aminobutyric Acid

**DOI:** 10.1128/genomeA.00552-15

**Published:** 2015-05-28

**Authors:** Ying Wang, Yu Wang, Chong Lang, Dongzhi Wei, Ping Xu, Jingli Xie

**Affiliations:** a State Key Laboratory of Bioreactor Engineering and Department of Food Science and Technology, East China University of Science and Technology, Shanghai, People’s Republic of China; b State Key Laboratory of Microbial Metabolism and School of Life Sciences and Biotechnology, Shanghai Jiao Tong University, Shanghai, People’s Republic of China; c Shanghai Collaborative Innovation Center for Biomanufacturing (SCICB), Shanghai, People’s Republic of China

## Abstract

*Lactobacillus curieae* CCTCC M 2011381^T^ is a novel species of the genus *Lactobacillus* and a gamma-aminobutyric acid producer that was isolated from stinky tofu brine. Here, we present a 2.19-Mb assembly of its genome, which may provide further insights into the molecular mechanisms underlying its beneficial properties.

## GENOME ANNOUNCEMENT

Gamma-aminobutyric acid (GABA), a 4-carbon nonproteinaceous amino acid found ubiquitously in nature, is associated with several well-characterized physiological functions (1–4). Many microorganisms can produce GABA, including bacteria, fungi, and yeasts, among which lactic acid bacteria (LAB) are the main GABA producers (5–8). GABA-producing LAB have been a focus of research in recent years, because LAB possess special physiological activities and are generally regarded as safe organisms, which have been extensively used in the food industry (6, 9–11).

*Lactobacillus curieae* CCTCC M 2011381^T^, which can produce GABA, is a novel species in the *Lactobacillus buchneri* clade isolated from stinky tofu brine in Shanghai, China (12). The strain was deposited at the China Center for Type Culture Collection (CCTCC) with the number 2011381 (type strain CCTCC M 2011381^T^ = S1L19^T^ = JCM 18524^T^). According to our primary test, this strain can ferment cow milk, soy beverage, and some other plant material, such as ginkgo seed beverage. Moreover, the fermentation of such materials with this strain can bring about an increase in the angiotensin-converting enzyme inhibitory activity (data not shown). However, *L. curieae* CCTCC M 2011381^T^ is not yet included in the catalogue of LAB used in food issued by the Chinese National Health and Family Planning Commission, since it was newly found and identified as a novel LAB strain. The genomic information is therefore significant and urgent for demonstrating its security in foods and expanding its potential usage.

Here, we present the first draft genome sequencing of *L. curieae* CCTCC M 2011381^T^, obtained by using the Illumina Solexa HiSeq 2000 instrument at the Beijing Genomics Institute (BGI), Shenzhen, China. Sequencing was performed with a paired-end library to produce 305 Mb of filtered sequences, representing about 140-fold coverage of the genome. The reads were assembled into 29 contigs (>1,000 bp) using the SOAPdenovo software (13, 14). Open reading frames (ORFs) were predicted from the assembled result using Glimmer (15–17) and translated by the EBI translation tool. The draft genome sequence of *L. curieae* CCTCC M 2011381^T^ was annotated with the NCBI Prokaryotic Genomes Automatic Annotation Pipeline (PGAAP). In addition, the contigs were searched against the Kyoto Encyclopedia of Genes and Genomes (KEGG) and Clusters of Orthologous Groups (COG) databases to annotate the gene descriptions.

The draft genome sequence comprises 2,185,962 bases with an average G+C content of 39.62%. This strain contains 1,957 protein-coding sequences covering 87.75% of the genome, of which 1,820 were annotated with clear biological functions and 310 were uncharacterized. Among them, 1,297 proteins have KEGG orthologs, and 1,200 proteins have COG classifications. It also harbors 56 tRNA-coding genes and 6 rRNA-coding operons. The genome sequence of *L. curieae* CCTCC M 2011381^T^ is a promising resource for further identifying the genes involved in beneficial effects, such as GABA production and food safety. In addition, it could also facilitate comparative genomics of *Lactobacillus* species.

### Nucleotide sequence accession number. 

This whole-genome shotgun project has been deposited at DDBJ/EMBL/GenBank under the accession number JTAL00000000. The version described in this paper is the first version, JTAL01000000.
